# Meeting of the Michigan Dental Association

**Published:** 1860-02

**Authors:** L. C. Whiting


### MEETING OF THE MICHIGAN DENTAL ASSOCIATION.

The fifth annual meeting of the Michigan Dental Association, convened in the city of Detroit, Tuesday evening January 10th, 1860, at 7 o'clock, and was called to order by the President, Dr. Porter of Ann Arbor.

The minutes of the last meeting were read by the Secretary and adopted.

On motion, Dr. G. H. Perine, of New York, Dr. A. Mayo, of Sandusky, Ohio; Dr. W. H. Atkinson, of Cleveland, O., were elected honorary members of the Association.

On motion, Drs. Porter, Atkinson, and Cahoon, were appointed a committee to prepare the order of business for this meeting.

On motion, Drs. Cahoon, Chittenden and Farmer, were appointed to examine candidates for admission to the Association.

On motion of Dr. Benedict, an invitation was extended to all practicing Dentists out of the State, and all students who are prepairing for the practice of Dentistry, to attend the meetings of the Association.

The Committee on candidates for membership, reported in favor of the following named persons who were unanimously elected: Dr. J. S. Corbin, of Ann Arbor; Dr. W. Y. Cleland, of Detroit; Dr. G. M. Cole, of Detroit; Dr. L. A. Rogers, of Grand Rapids.

The Association then proceeded to the election of officers for the ensuing year.

*President,* Dr. C. S. Chittenden, of Hamilton, Canada West;

*Vice President*, Dr. Wm. Cahoon, of Detroit; *Secretary and Treasurer*, Dr. L. C. Whiting; *Executive, Examining and Publishing Committee*, Drs. H. Benedict, J. H. Farmer, T. H. Farmer, and T. A. White.

Meeting then adjourned to 9 o'clock Wednesday morning.

WEDNESDAY MORNING.

Dr. Porter on retiring from the Chair made a short but elegant address.

Dr. Chittenden on being conducted to the Chair made a few remarks, thanked the Society for the honor confered upon him, and should do the best he could to serve them.

Committee reported order of business which was adopted.

Several essays were read on difficult dentition, the effects of diseased teeth and gums on the general health of the system, after which a discussion was entered into which elicited much interesting information.

Adjourned to meet again at 2 o'clock P. M.

AFTERNOON SESSION.

Meeting convened at 2 o'clock, P. M., and the discussions continued, with reading correspondence, &c.

On motion of Dr. Porter, a committee of six was appointed to write essays on the different subjects for discussions at the next meeting.

The following were the committee with the subjects on which they were appointed to write:

Dr. Whiting, on the effects of Diseased Gums and Teeth on the general health.

Dr. Porter, on Difficult Dentition.

Dr. Farmer, on Neuralgia and Rheumatism.

Dr. Mansfield, on Mechanical Dentistry.

Dr. Cahoon, on Dental Etiquette and Fees.

Dr. Chittenden, Second Dentition.

On motion of Dr. Porter, *Resolved*, that the delegates to the National Society be elected by ballot.

On vote, Dr. Whiting and Cahoon, of Detroit, were elected delegates to the National Society.

On motion of Dr. Benedict, *Resolved*, that the delegates have power to appoint substitutes in case they can not attend.

On motion of Dr. Porter, *Resolved*, that one half the traveling expenses of delegates be paid by the Association.

On motion of Dr. Porter, *Resolved*, that the dues remitted to members heretofore be paid, in order to raise funds to pay expenses of delegates.

On motion of Dr. Mansfield, no member of this Society shall take a student into his office, and engage to teach him the practice of either department of Dentistry, in less time than two years; and all such students, during their pupilage, shall have the privilege of attending the meetings of the Society, but shall take no part in its deliberations.

On motion, *Resolved*, that Mechanical Dentistry be the subject of discussion in the evening.

Moved that Dr. Atkinson of Cleveland now read an address to the Association.

After the address Society adjourned to 7 o'clock.

EVENING SESSION.

Moved that when the Association adjourns, it does so to meet at Ann Arbor, the second Tuesday of January 1861.

On motion, *Resolved*, that a vote of thanks be given those members of the profession, who have sent their fee bills for the benefit of this Association.

Moved the Association adjourn at 9 o'clock.

The evening was spent in interesting conversation on Mechanical Dentistry.

L. C. WHITING, *Sec'y.*

